# Ventricular Repolarization is Associated with Cognitive Function, but Not with Cognitive Decline and Brain Magnetic Resonance Imaging (MRI) Measurements in Older Adults

**DOI:** 10.3390/jcm9040911

**Published:** 2020-03-26

**Authors:** Michelle H. Zonneveld, Raymond Noordam, Jeroen van der Grond, Behnam Sabayan, Simon P. Mooijaart, Peter W. Mcfarlane, J. Wouter Jukema, Stella Trompet

**Affiliations:** 1 Department of Internal Medicine, Section of Gerontology and Geriatrics, Leiden University Medical Center, 2333 ZA Leiden, The Netherlands; M.H.Zonneveld@lumc.nl (M.H.Z.); S.P.Mooijaart@lumc.nl (S.P.M.); S.Trompet@lumc.nl (S.T.); 2 Department of Radiology, Leiden University Medical Center, 2333 ZA Leiden, The Netherlands; J.van_der_Grond@lumc.nl; 3 Feinberg School of Medicine, Northwestern University, Chicago, IL 60611, USA; Behnam.Sabayan@northwestern.edu; 4 Institute of Health and Wellbeing, University of Glasgow, Glasgow G31 2ER, UK; Peter.Macfarlane@glasgow.ac.uk; 5 Department of Cardiology, Leiden University Medical Center, 2333 ZA Leiden, The Netherlands; J.W.Jukema@lumc.nl

**Keywords:** older adults, cardiovascular disease, cognitive dysfunction, MRI, prolonged QT interval

## Abstract

We aimed to investigate the cross-sectional and longitudinal associations of electrocardiogram (ECG)-based QT, QTc, JT, JTc, and QRS intervals with cognitive function and brain magnetic resonance imaging (MRI) measurements in a cohort of older individuals at increased risk for cardiovascular disease, but free of known arrhythmias. We studied 4627 participants (54% female, mean age 75 years) enrolled in the Prospective Study of Pravastatin in the Elderly at Risk (PROSPER). Ten-second ECGs were conducted at baseline. Cognitive function was tested at baseline and repeated during a mean follow-up time of 3.2 years. Structural MRIs were conducted in a subgroup of 535 participants. Analyses were performed with multivariable (repeated) linear regression models and adjusted for cardiovascular risk-factors, co-morbidities, and cardiovascular drug use. At baseline, longer QT, JT, JTc—but not QTc and QRS intervals—were associated with a worse cognitive performance. Most notably, on the Stroop Test, participants performed 3.02 (95% CI 0.31; 5.73) seconds worse per standard deviation higher QT interval, independent of cardiovascular risk factors and medication use. There was no association between longer ventricular de- or repolarization and structural brain measurements. Therefore, specifically ventricular repolarization was associated with worse cognitive performance in older individuals at baseline but not during follow-up.

## 1. Introduction

The incidence of dementia has increased in the last decades as a consequence of a growing number of older individuals [1]. In recent years, the relationship between cardiac function and cognitive impairment has been studied to a significant extent [2,3,4,5]. Several epidemiological and clinical studies have placed emphasis on cardiovascular disease as a contributing factor to the pathogenesis of various brain disorders, such as Alzheimer’s disease (AD) and dementia [2,6,7,8,9]. More specifically, cardiac function markers, such as heart-rate variability and left ventricular hypertrophy, have been associated with worsened cognitive function and abnormalities within the brain, such as white matter hypertensities (WMH), as seen in dementia [6,10,11,12,13]. Studies using magnetic resonance imaging (MRI) have demonstrated that a high microbleed count, as a marker of diffuse vascular brain damage, is associated with an increased risk for cognitive decline and dementia [14]. Other well-known cardiovascular risk factors that contribute to brain diseases, such as dementia, include hypertension, hypercholesteremia, diabetes, obesity, and smoking [7,15,16].

The electrocardiogram (ECG) is an easily-measured tool to record complex electrophysiological alterations during each cardiac cycle [17]. A study from 2010 reports that coronary heart disease is more likely to affect ventricular repolarization than ventricular excitation [3]. In relation to cognition, individuals with a wider QRS-T angle have an accelerated decline in overall cognitive functioning [18]. Another study investigating relationships between cognitive function and non-invasive, repeatable cardiac parameters in older adults report an inverse association between Mini-Mental State Examination (MMSE) score and QT interval in patients with mild cognitive impairment (MCI) [2]. These findings suggest a relationship between measures of pre-clinical cardiac pathology involving prolonged QT interval, as a measure of prolonged ventricular repolarization, and future cognitive decline. However, to the best of our knowledge the association between prolonged ventricular de- and repolarization and cognitive decline in an older population without severe cognitive problems remains unexplored.

In this study, we assessed the association of the QT, QTc, JT, JTc, and QRS intervals with cognition at baseline, cognitive decline during follow-up, and MRI-based structural brain status in a cohort of older individuals at increased risk for cardiovascular disease.

## 2. Materials and Methods

### 2.1. Ethical Approval

The original Prospective Study of Pravastatin in the Elderly at Risk (PROSPER) was approved by the Medical Ethics Committees of the three collaborating centers and complied with the Declaration of Helsinki. All participants gave written informed consent in accordance with the Declaration of Helsinki.

### 2.2. Study Design

The data used for this study was obtained from the Prospective Study of Pravastatin in the Elderly at Risk (PROSPER). This large, multicenter randomized clinical trial investigated the safety and efficacy of pravastatin in older participants with preexisting or at high risk of cardiovascular disease in three countries (the Netherlands, Scotland, Ireland). The participants—5804 men and women aged 70–82 years—were enrolled, and had a follow-up time of 3.2 years on average. Participants with the following conditions were not recruited in the PROSPER study: congestive heart failure, significant arrhythmia, cognitive impairment (Mini-Mental Score Examination score <24). The inclusion and exclusion criteria of PROSPER have been described in detail elsewhere [19,20]. Participants in the PROSPER study were reviewed approximately every three months. MRIs were conducted in Dutch participants who completed the trial only.

### 2.3. Study Participants

In the present study, the following exclusion criteria were applied to the participants from the PROSPER study: QRS interval duration ≥120 ms; atrial fibrillation on baseline ECG; non-sinus rhythms; extreme heart rates (<40 or >120 BPM). Non-sinus rhythms included premature ventricular or atrial contractions, ectotopic atrial rhythm, atrial flutter, supraventricular arrhythmia, and other arrhythmias. Additionally, participants with incomplete ECG measurements at baseline and missing cognitive function measurements at baseline or during follow-up were excluded. Both participants from pravastatin and placebo groups were included, as it was shown previously that pravastatin does not affect cognitive function [19].

### 2.4. Data Collection

#### 2.4.1. ECG Measurements

The resting ECGs were measured at baseline only. A 12-lead Burdick Eclipse 850i electrocardiograph was used to record ECGs annually; these were transmitted electronically to the ECG core laboratory at Glasgow Royal Infirmary for storage and review [21]. All ECGs were analyzed using the University of Glasgow automated ECG analysis program. Ten-second ECG recordings were completed in a resting, supine position. The PR, QRS, and QT intervals were measured for each ECG. QTc interval is the corrected version of the QT interval and was calculated using Hodges’ formula [22]. The JT interval was defined as the length of the QT interval minus the duration of the QRS complex. The JTc interval is the heart rate-corrected version of the JT interval, and was also calculated using Hodges’ formula. The JT interval was also used as it has been proposed that this measurement provides a more appropriate indication of ventricular repolarization than QT interval [17].

#### 2.4.2. Cognitive Function Measurements

The cognitive function tests were conducted at baseline, after 9, 18, and 30 months, and at the end of the study [23]. The timepoint at the end of the study ranged from 36 to 48 months [10]. The MMSE was used as a baseline measurement to represent global cognitive function, based on a score between 0 and 30 points. Participants with a score below 24 points on the MMSE were excluded. Four other neuropsychological performance tests were used to assess cognitive function. Reaction time and selective attention were assessed using the Stroop Test, where the outcome variable is response time between 0 and 100 s. In the Stroop Test, a higher score indicates a worse performance. General cognitive processing speed was measured by the Letter–Digit Coding Test. The outcome variable was the total number of correct digits entered in 60 s. Here, a higher score indicates a better performance [10]. The Picture–Word Learning Test was used to assess immediate and delayed memory. The outcome variable consists of the total number of correctly recalled pictures over 3 attempts and the number of pictures recalled during delayed recall. Here, a higher score indicates better performance (see the online supplement for additional procedural details concerning the cognitive function tests).

#### 2.4.3. MRI Measurements

Participants (*n* = 535) from the Netherlands underwent an MRI scan of the brain at baseline. Procedural details of the MRI scanning have been reported elsewhere [24]. The following measurements were obtained: white matter hyperintensities including total lesion, subcortical and periventricular volume; brain atrophy, including intracranial and parenchymal volume; grey matter volume; microbleeds, including deep white matter microbleeds.

#### 2.4.4. Covariates

For each participant, an extensive medical history was obtained during a 10-week screening period using routine care data. Education, medication use, such as anti-hypertensive medication, smoking status, alcohol intake were evaluated as previously described using a medical inventory [20]. At 6 and 9 months, a fasting venous blood sample was drawn to measure lipid and lipoprotein profiling [20]. History of diabetes mellitus was defined as fasting blood glucose ≥7 mmol/L or self-reported. Data on history of transient ischemic attack, stroke, or myocardial infarction were provided by the participant’s general practitioner.

### 2.5. Statistical Analysis

Baseline characteristics of the study participants are reported as mean (standard deviation) for continuous variables and number (percentage) for categorical variables. The following ECG measurements were used and standardized (mean = 0, standard deviation = 1): QT interval; QTc interval; JT interval; JTc interval; QRS interval. Both the QT and QTc intervals, as well as JT and JTc intervals, are reported in order to see the difference between correcting for heart rate before and during the regression analysis. The associations between the measures of ventricular de-/repolarization and measures of cognitive function were studied using multivariable linear regression analyses.

In order to determine the cross-sectional associations between ECG measurements (determinant), cognitive functioning at baseline and brain status at baseline (outcomes), multivariable linear regression models were reported using a beta coefficient per standard deviation with 95% confidence interval. A linear mixed methods model was used to determine the longitudinal association between ECG measurements and cognitive decline. The interaction term between time and the ECG measurement was used to determine the association.

The cross-sectional and longitudinal analyses were performed in two steps. At first, the multivariable linear regression analyses were adjusted for age, sex, country, and heart rate (QT, JT, and QRS intervals only, as the QTc and JTc intervals is already corrected for heart rate). This approach was denoted as the minimally adjusted model. In the second step, the analyses were further adjusted for: alcohol intake per week; smoking; educational level, BMI; serum cholesterol; diabetes mellitus; systolic blood pressure; antihypertensive medication including diuretics, ACE-inhibitors, ACE II-inhibitors, beta-blockers, calcium channel blockers, vasodilators, antidepressants, anticholinergic medication, antiarrhythmic medication (denoted at the fully adjusted model) [24]. All results were presented as the additional decline per standard deviation increase in ECG measures together with the accompanying 95% confidence interval.

In addition, we performed a number of stratified analyses to study the potential of possible effect modification, notably by sex, history of cardiovascular disease, history of myocardial infarction, pro-natriuretic peptide concentration above and below the median concentration, use of beta-blockers, and diagnosis of diabetes mellitus. Possible effect modification on a multiplicative scale was studied by including an interaction term in the linear mixed methods regression model, where a two-sided *p*-value < 0.05 was considered statistically significant.

## 3. Results

### 3.1. Baseline Characteristics

The PROSPER trial included 5804 participants. After excluding patients with missing ECG data (*n* = 148, 2.5%), non-sinus rhythms (*n* = 467, 8.0%), and a QRS duration > 120 ms (*n* = 562, 9.7%), 4627 participants remained and were included in the analysis (see Supplementary Figure S1 in supplement for patient inclusion flow diagram).

Characteristics of the study population at baseline, including cognitive function and brain status, are reported in Table 1. Over half of the participants were female (*n* = 2480, 53.6%) and the mean age of all the cohort was 75.2 years. Approximately a third were current smokers (*n* = 1255, 27.1%) and almost half had a history of cardiovascular disease (*n* = 1996, 43.1%). The large majority of patients used antihypertensive medication (*n* = 3425, 74.0%). The mean score on the Stroop Test was 65.5 s (median = 65.5 s, SD = 26.1).

Of the 885 participants from the Netherlands, 440 participants had undergone an MRI scan (49.7%), of which 45% were female (*n* = 198). The median total lesion volume was 1.6 mL (median = 1.6, Interquartile range (IQR) = 0.5–5.5) and the mean percentage atrophy was 26.1% (mean = 26.1, SD = 3.1). 104 participants had at least 1 microbleed (*n* = 104, 2.2%) and 28 participants had at least 1 deep white matter microbleed (*n* = 28, 0.6%).

### 3.2. Cognitive Function Analyses

In Table 2, the associations between the measures of ventricular depolarization and repolarization and cognitive function at baseline are shown. In the minimally adjusted model, a 1 standard deviation increase in the QT interval (95% CI 1.48; 3.72) is associated with an increase of 2.60 s on the Stroop Test. Likewise, each 1 standard deviation increase in QTc, JT, and JTc intervals was associated with a worse performance on the Letter–Digit Coding Test.

**Figure 1:**
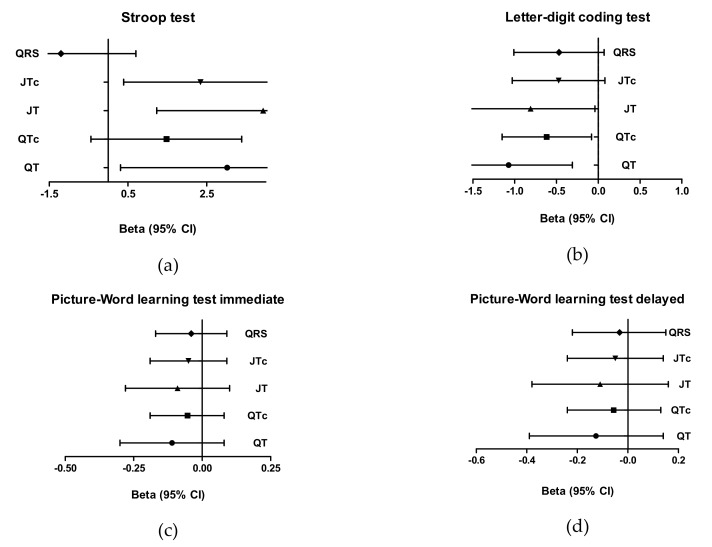
Associations between QT, QTc, JT, JTc, and QRS intervals, and cognitive tests as Beta (95% CI) (fully adjusted model). (**a**) Association between QT, QTc, JT, JTc, QRS intervals and the Stroop Test. (**b**) Association between QT, QTc, JT, JTc, QRS intervals and the Letter–Digit Coding Test. (**c**) Association between QT, QTc, JT, JTc, QRS intervals and the Picture–Word Learning Test immediate. (**d**) Association between QT, QTc, JT, JTc, QRS intervals and the Picture–Word Learning Test delayed.

After additional adjustments, a one standard deviation increase in the QT interval (95% CI 0.31; 5.73) is associated with an increase of 3.02 s on the Stroop Test. The pattern in associations between ECG measurements and cognitive tests remained similar (Figure 1).

The results of the longitudinal associations are displayed in Table 3. In both the minimally adjusted model and fully adjusted model, one standard deviation increase in ventricular depolarization and repolarization at baseline was not associated with a faster decline in performance on cognitive test over time. The effect sizes are minimal. Supplementary Figures S1–S3 show the results of the stratifications of the linear regressions in order to study possible effect modification. After performing sensitivity analyses on the fully adjusted models, we did not find evidence of effect modification.

### 3.3. Brain MRI Measurements

Table 4 displays the results of the cross-sectional associations at baseline between brain status parameters and measures of ventricular de- and repolarization. In the minimally adjusted model (see supplementary data), each standard deviation increase of specifically the QT, JT, and JTc intervals were associated with an increase in the number of microbleeds (Beta = 0.36, 95% CI: 0.02, 0.70; beta = 0.41, 95% CI: 0.08, 0.74; beta = 0.34, 95% CI: 0.10, 0.58). The associations attenuated after adjustment for considered confounding factors.

## 4. Discussion

This study explored the associations between the extent of ventricular depolarization and repolarization, and various domains of cognitive functioning and brain MRI measures in a cohort of older individuals at increased risk for cardiovascular disease. Cross-sectionally, we found that prolonged ECG intervals were associated with a worse performance in all cognitive domains. The association remained after adjusting for multiple possible confounders. We did not observe further associations between the measures of ventricular de/repolarization at baseline and cognitive decline during the study, nor did we find evidence of effect modification. In relation to brain MRI measures, at baseline in the minimally adjusted model we found a small cross-sectional association between prolonged ventricular de- and repolarization and an increased number of microbleeds. This association did not remain after additional adjustments.

Previous cross-sectional studies have also reported significant associations between ECG intervals in the QRST complex and cognitive function. For example, Lucas et al. report higher levels of T-wave non-dipolar voltage to be significantly associated with poorer global cognitive performance in older participants [3]. Similarly, Stenfors et al. report that a lower QT variability index (QTVI), which is a measure of myocardial repolarization patterns in time, is to be associated with better performance on both the Stroop Test and Letter–Digit Coding Test [25,26]. However, the latter study was conducted in healthy, working adults; thus, limiting the comparison to the present study. In both studies, the association remained after adjusting for demographic factors. Another study found Alzheimer’s disease (AD) and mild cognitive impairment were more frequent in individuals with higher QT dispersion [2].

The strongest effect was found between the JT interval, representing ventricular repolarization, and cognitive performance, followed by the association between the QT interval, representing both ventricular de- and repolarization, and cognition. There are several potential explanations for these associations. First, a prolonged ventricular de- and repolarization period and cognitive decline may both reflect cardiovascular damage and thus share a common cause. Previous research has demonstrated the presence of age-related decline in autonomic function and thus decline in control of duration of de- and repolarization [27]. This study also showed a clear association between increasing age and higher QT variation index (QTVI), which is in line with earlier studies [2,28]. Second, an increase in QTVI has also been connected to anxiety and depression, which are major risk factors for cognitive decline and dementia [29,30,31]. However, in the present study, data on depression, stress or burnout was not collected and therefore we cannot exclude the influence of the aforementioned on the respective ECG intervals. Last, a persistent prolonged QT interval could be linked to a genetic predisposition, medication use, hypokalemia, or hypomagnesemia. In case of drug-induced QT interval prolongation, the prolongation of the action potential is either the result of an increase in inward current or a decrease in outward current. The hallmark of this type of long QT is the blockade of the rapid delayed rectifier potassium current, also called IKr [32]. This channel is primarily responsible for repolarization, and a blockade causes a delay in rapid repolarization of the action potential, which is reflected by QT prolongation [32]. This could explain why in our results the largest effect size (specifically with the Stroop Test) was seen in either the QT or JT intervals, as the JT interval solely reflects the repolarization phase. Results from another study concur with our findings and suggest that ventricular repolarization may be more sensitive to changes resulting from coronary heart disease than ventricular depolarization [3].

We also observed a cross-sectional association between the prolongation of the QT, JT, and JTc intervals and the number of microbleeds. This association has not been reported previously. However, other studies investigating prolongation of ventricular de/repolarization and brain imaging correlations did find an association between prolonged QTc and global and temporal atrophy, indicated by the bicaudate ratio [33]. A possible explanation could be that patients with a higher degenerative damage have a greater impairment of the cerebral autonomic network, which is then reflected by the prolongation of QTc. Thus, cerebrovascular load seems to be associated with a prolongation of QTc.

This study has several strengths: its prospective multicenter design; large sample size (more than 4000 participants); and the use of several cognitive tests assessing various domains of cognitive function. We were also able to demonstrate that the results are independent of cardiovascular risk-factors and medication use. Furthermore, ECGs are potentially a suitable tool for detection of cardiovascular disease and cognitive decline in clinical practice due to the low costs and non-invasive nature. One limitation of this study is the limited follow-up time in PROSPER. As this was relatively short (3.2 years on average), the cognitive test scores may not reflect cognitive decline over a longer time period accurately and this may explain the fact that a longitudinal association was not found over 3.2 years. We also did not consider adjusting our type-1 error rate by correction for multiple testing given the hypothesis-testing nature of our study and the interrelations of the different analyses conducted. Additionally, the participants in this study either were at high risk, or had a history of cardiovascular disease, hampering the generalizability of our findings to a healthy, older population. Nevertheless, a considerable proportion of the older adults have cardiovascular pathologies and our findings were independent of cardiovascular risk-factors and use of medication. Another limitation is the relatively small magnitude of associations. This could be attributable to the PROSPER exclusion criteria at baseline (<24 points on MMSE), leading to participants with relatively preserved cognitive function at baseline.

In conclusion, longer ventricular de- and repolarization as reflected by the QT, JT, and JTc intervals is associated with a steeper decline in cognitive function, independent of cardiovascular risk factors and use of medication. The JT interval, representing ventricular repolarization, had an especially strong effect on cognitive performance. Older adults with longer QT, JT, or JTc intervals may be identified as a risk group for future cognitive impairment. Future research should aim to further elucidate causality and examine this association with a longer follow-up time.

## Figures and Tables

**Table 1:** Demographic and clinical characteristics of study population (*n* = 4627).

Age, y, mean (SD)	75.2 (3.3)
Female, *n* (%)	2480 (53.6)
Age left school, y, mean (SD)	15.1 (2.0)
Current smoker, *n* (%)	1255 (27.1)
**Cardiovascular risk factors**
History of CVD, *n* (%)	1996 (43.1)
History of stroke or TIA, *n* (%)	511 (11.0)
History of MI, *n* (%)	540 (11.7)
Serum cholesterol, mmol/L, mean (SD)	5.7 (0.9)
Body mass index, kg/m^2^, mean (SD)	26.8 (4.2)
Diabetes mellitus, *n* (%)	474 (10.2)
Antihypertensive therapy, *n* (%)	3425 (74.0)
Pravastatin treatment, *n* (%)	2309 (50.0)
SBP, mmHg, mean (SD)	154.7 (21.6)
DBP, mmHg, mean (SD)	83.8 (11.4)
**Electrocardiogram measurements**
QT duration, ms, mean (SD)	413.0 (35.6)
QTc duration, ms, mean (SD)	424.9 (25.5)
JT duration, ms, mean (SD)	319.6 (34.3)
JTc duration, ms, mean (SD)	332.5 (25.0)
QRS duration, ms, mean (SD)	93.4 (11)
**Cognitive function**	
Stroop Test, seconds, mean (SD) ^1^	65.6 (26.1)
LDCT, digits coded, mean (SD) ^2^	23.3 (7.9)
PLTi, pictures remembered mean (SD) ^3^	9.3 (1.9)
PLTd, pictures remembered, mean (SD) ^3^	10.1 (2.6)
**Brain MRI measurements**	
White matter hypertensities ^4^
Total lesion volume, ml, median (IQR)	1.6 (0.5–5.5)
Subcortical, mL/y, median (IQR)	0.5 (0.1–1.4)
Periventricular, mL/y, median (IQR)	1 (0.3–3.9)
Brain atrophy ^5^
Intracranial volume, ml, mean (SD)	1401 (144.5)
Parenchymal volume, ml, mean (SD)	1034.3 (26.1)
Of % atrophy (ICV-Par/ICV ×100), mean (SD)	26.1 (3.1)
Grey matter volume, mL, median (IQR) ^6^	23.2 (20.6–29.2)
Participants with microbleeds, *n* (%) ^7^	104 (2.2)
Participants with deep white matter microbleeds, *n* (%)	28 (0.6)

Abbreviations: CVD = cardiovascular disease; TIA = transient ischemic attack; MI = myocardial infarction; LDCT = Letter-Digit Coding Test; PLTi = Picture–Word Learning Test immediate; PLTd = Picture–Word Learning Test delayed; ICV = intracranial volume; Par = parenchymal volume; IQR = interquartile range. ^1^ = performed by 4293 participants, ^2^ = performed by 4327 participants, ^3^ = performed by 4359 participants, ^4^ = conducted in 445 participants, ^5^ = conducted in 440 participants, ^6^ = conducted in 221 participants, ^7^ = conducted in 430 participants.

**Table 2:** Cross-sectional associations between measures of ventricular de- and repolarization and cognitive function at baseline.

	QT (per SD)	QTc (per SD)	JT (per SD)	JTc (per SD)	QRS (per SD)
Cognitive Test	Beta (95% CI)	Beta (95% CI)	Beta (95% CI)	Beta (95% CI)	Beta (95% CI)
**Minimally adjusted ^1^**					
Stroop, s	2.60 (1.48; 3.72)	1.13 (0.36; 1.91)	2.22 (1.10; 3.34)	1.00 (0.20; 1.79)	0.74 (–0.06; 1.53)
LDCT, digits coded	–0.97 (–1.31; –0.63)	–0.49 (–0.73; –0.25)	–0.67 (–1.01; –0.34)	–0.34 (–0.57; –0.10)	–0.51 (–0.75; –0.26)
PLTi, pictures remembered	–0.14 (–0.22; –0.06)	–0.0 (–0.005; 0.00)	–0.09 (–0.17; –0.01)	–0.04 (–0.10; 0.01)	–0.08 (–0.14; –0.02)
PLTd, pictures remembered	–0.17 (–0.28; –0.06)	–0.07 (–0.14; 0.01)	–0.12 (–0.23; 0.00)	–0.04 (–0.12; 0.04)	–0.10 (–0.18; –0.02)
**Fully adjusted ^2^**
Stroop, s	3.02 (0.31; 5.73)	1.48 (–0.44; 3.39)	3.93 (1.23; 6.64)	2.34 (0.39; 4.30)	–1.20 (–3.11; 0.70)
LDCT, digits coded	–1.07 (–1.84; –0.31)	–0.62 (–1.15; –0.08)	–0.81 (–1.58; –0.04)	–0.47 (–1.03; 0.08)	–0.47 (–1.01; 0.07)
PLTi, pictures remembered	–0.11 (–0.30; 0.08)	–0.05 (–0.19; 0.08)	–0.09 (–0.28; 0.10)	–0.05 (–0.19; 0.09)	–0.04 (–0.17; 0.09)
PLTd, pictures remembered	–0.13 (–0.39; 0.14)	–0.06 (–0.24; 0.13)	–0.11 (–0.38; 0.16)	–0.05 (–0.24; 0.14)	–0.03 (–0.22; 0.15)

Abbreviations: SD = standard deviation; 95% CI = 95% confidence interval; LDCT = Letter–Digit Coding Test; PLTi = Picture–Word Learning Test immediate; PLTd = Picture–Word Learning Test delayed. ^1^ = adjusted for age, sex, country, heart rate (QT, JT, and QRS only), ^2^ = adjusted for age, sex, country, heart rate (QT, JT, and QRS only), alcohol intake per week, smoking, education level, BMI, serum cholesterol, antihypertensive medication, anticholinergic medication, antiarrhythmic medication, antidepressants, diabetes mellitus, systolic blood pressure.

**Table 3:** Longitudinal associations between measures of ventricular de- and repolarization and cognitive function.

	QT (per SD)	QTc (per SD)	JT (per SD)	JTc (per SD)	QRS (per SD)
Cognitive Test	Estimate (95% CI)	Estimate (95% CI)	Estimate (95% CI)	Estimate (95% CI)	Estimate (95% CI)
**Minimally adjusted ^1^**	
Stroop, s	–0.03 (–0.3; 0.24)	–0.02 (–0.29; 0.25)	–0.07 (–0.33; 0.20)	–0.06 (–0.33; 0.20)	0.10 (–0.17; 0.37)
LDCT, digits coded	0.04 (–0.03; 0.12)	0.05 (–0.03; 0.12)	0.05 (–0.03; 0.12)	0.05 (–0.02; 0.13)	–0.01 (–0.08; 0.06)
PLTi, pictures remembered	0.00 (–0.02; 0.02)	0.006 (–0.01; 0.03)	0.00 (–0.02; 0.02)	0.01 (–0.01; 0.03)	0.00 (–0.02; 0.02)
PLTd, pictures remembered	0.00 (–0.03; 0.02)	–0.007 (–0.04; 0.02)	0.00 (–0.03; 0.03)	0.00 (–0.03; 0.03)	–0.006 (–0.04; 0.02)
**Fully adjusted ^2^**	
Stroop, s	0.02 (–0.61; 0.65)	–0.13 (–0.77; 0.52)	–0.15 (–0.79; 0.49)	–0.36 (–1.02; 0.29)	0.44 (–0.18; 1.06)
LDCT, digits coded	0.08 (–0.08; 0.24)	0.02 (–0.15; 0.19)	0.09 (–0.08; 0.25)	0.02 (–0.15; 0.19)	0.01 (–0.15; 0.17)
PLTi, pictures remembered	–0.02 (–0.07; 0.03)	–0.03 (–0.08; 0.02)	–0.02 (–0.07; 0.03)	–0.04 (–0.09; 0.01)	0.01 (–0.04; 0.05)
PLTd, pictures remembered	0.00 (–0.07; 0.07)	–0.03 (–0.10; 0.04)	0.00 (–0.07; 0.07)	–0.04 (–0.11; 0.04)	0.00 (–0.07; 0.07)

Abbreviations: SD = standard deviation; 95% CI = 95% confidence interval; LDCT = Letter–Digit Coding Test; PLTi = Picture–Word Learning Test immediate; PLTd = Picture–Word Learning Test delayed. ^1^ = adjusted for age, sex, country, heart rate (QT, JT, and QRS only), ^2^ = adjusted for age, sex, country, heart rate (QT, JT, and QRS only), alcohol intake per week, smoking, education level, BMI, serum cholesterol, antihypertensive medication, anticholinergic medication, antiarrhythmic medication, antidepressants, diabetes mellitus, systolic blood pressure.

**Table 4:** Cross-sectional associations between measures of ventricular de- and repolarization and brain MRI measurements at baseline.

	QT (per SD)	QTc (per SD)	JT (per SD)	JTc (per SD)	QRS (per SD)
Brain MRI Measurements	Beta (95% CI)	Beta (95% CI)	Beta (95% CI)	Beta (95% CI)	Beta (95% CI)
**Fully adjusted ^1^**
White matter hypertensities					
Total lesion volume, mL/y	–0.21 (–2.87; 2.44)	–0.01 (–1.81; 1.79)	–0.45 (–2.82; 1.93)	–0.19 (–1.85; 1.46)	0.41 (–1.25; 2.07)
Subcortical, mL/y	–0.08 (–0.60; 0.44)	–0.04 (–0.39; 0.31)	–0.13 (–0.59; 0.34)	–0.07 (–0.40; 0.25)	0.09 (–0.24; 0.42)
Periventricular, mL/y	–0.13 (–2.42; 2.15)	0.03 (–1.52; 1.58)	–0.32 (–2.37; 1.73)	–0.12 (–1.55; 1.31)	0.32 (–1.11; 1.75)
Brain atrophy
Intracranial volume, mL/y	–4.0 (–44.3; 36.3)	4.8 (–23.0; 32.6)	–5.9 (–41.8; 30.1)	2.1 (–23.4; 27.6)	3.9 (–21.2; 29.0)
Parenchymal volume, mL/y	–3.7 (–34.3; 26.8)	3.2 (–17.9; 24.3)	–6.2 (–33.5; 21.1)	–0.5 (–18.9; 19.8)	4.8 (–14.3; 23.8)
Of % atrophy (ICV-Par/ICV ×100)	0.04 (–1.05; 1.14)	0.02 (–0.72; 0.76)	0.11 (–0.87; 1.08)	0.07 (–0.61; 0.74)	–0.11 (–0.79; 0.57)
Grey matter volume, mL/y	–1.12 (–3.19; 0.95)	–0.68 (–2.06; 0.71)	–1.19 (–3.03; 0.64)	–0.81 (–2.10; 0.49)	0.48 (–0.96; 1.91)
Number of microbleeds	0.33 (–0.28; 0.94)	0.19 (–0.21; 0.59)	0.37 (–0.17; 0.91)	0.23 (–0.14; 0.60)	–0.14 (–0.51; 0.23)
Number of deep white matter microbleeds	0.01 (–0.08; 0.10)	0.01 (–0.05; 0.07)	0.02 (–0.06; 0.10)	0.02 (–0.03; 0.08)	–0.02 (–0.08; 0.03)

^1^ = adjusted for age, sex, country, heart rate (QT, JT, and QRS only), alcohol intake per week, smoking, education level, BMI, serum cholesterol, antihypertensive medication, anticholinergic medication, antiarrhythmic medication, antidepressants, diabetes mellitus, systolic blood pressure.

## Supplementary Materials

The following are available online at https://www.mdpi.com/2077-0383/9/4/911/s1, Methods S1: Supplementary methods of cognitive function testing, Figure S1: Flow diagram of patient inclusion, Table S1: Cross-sectional association at baseline between measures of ventricular de- and repolarization and brain MRI status parameters (minimally adjusted model), Figure S2: Stratifications for QT and QTc intervals and the Stroop Test (fully adjusted model), Figure S3: Stratifications for JT and JTc intervals and the Stroop Test (fully adjusted model), Figure S4. Stratifications for QRS interval and the Stroop Test (fully adjusted model).
