# Supplementary material for: Ventricular Repolarization is Associated with Cognitive Function, but Not with Cognitive Decline and Brain Magnetic Resonance Imaging (MRI) Measurements in Older Adults
Source: J Clin Med. 2020 Mar 26;9(4):911. doi: 10.3390/jcm9040911 (PMC7230741; doi:10.3390/jcm9040911)
Supplement: Supplementary file 1 [file jcm-09-00911-s001.pdf]

Methods S1. Supplementary Methods of Cognitive Function Testing

As previously described, three different cognitive tests were used to evaluate cognitive performance: the Stroop Test; the Letter–Digit Coding Test; the Picture–World Learning Test, both immediate and delayed. Due to the international setup of the Prospective Study of Pravastatin in the Elderly at Risk (PROSPER) study, these tests were selected on the fact that they are not sensitive to language nor cultural differences. Thus, pictures, colors, and digits were used as opposed to words.

The Stroop Test, also called the Stroop Color and Word Test, was used to test selective attention. The test consists of three parts, each with 40 stimuli; the subject is asked to name, as quickly as possible, the color names, colored patches, and color names printed in incongruous colored ink. This means that if the word "blue" is printed in green letters, the subject is expected to say "green". It was not necessary to use parallel versions of the Stroop Test, as the location of the many color names on the test sheets cannot be remembered. In part 3, performance is largely decided by the subject's ability to tackle cognitive interference, such as disregarding irrelevant information. In PROSPER, the score for part 3 was used to determine selective attention.

The Letter–Digit Coding Test is a modified version of the Symbol–Digits Modalities Test, which is used for measuring the speed of processing of general information. In general, the test draws upon several processes simultaneously, such as motor functions, visual memory, and perception. Following the key, consisting of nine consonants in a random order presented at the top of the test sheet, the subject fills in digits near letters as fast as possible. On the test sheet, there are 125 randomly distributed letters from the key. Ceiling effects are ruled out, as a subject will typically make only 15–30 correct entries. The correct key at the top of the test sheet was different for every version of the test.

The Picture–Word Learning Test is derived from the Groningen Fifteen Words Test. The following procedure was carried out three times: a total of fifteen pictures were presented consecutively, two seconds per pictures, after which the subject was asked to recall as many pictures as possible. After 20 minutes, delayed recall was tested. As previously mentioned, pictures (instead of words) were chosen to prevent language problems. The main outcome was the total number of correctly recalled pictures over the three learning trials and the number of pictures recalled at delayed recall.

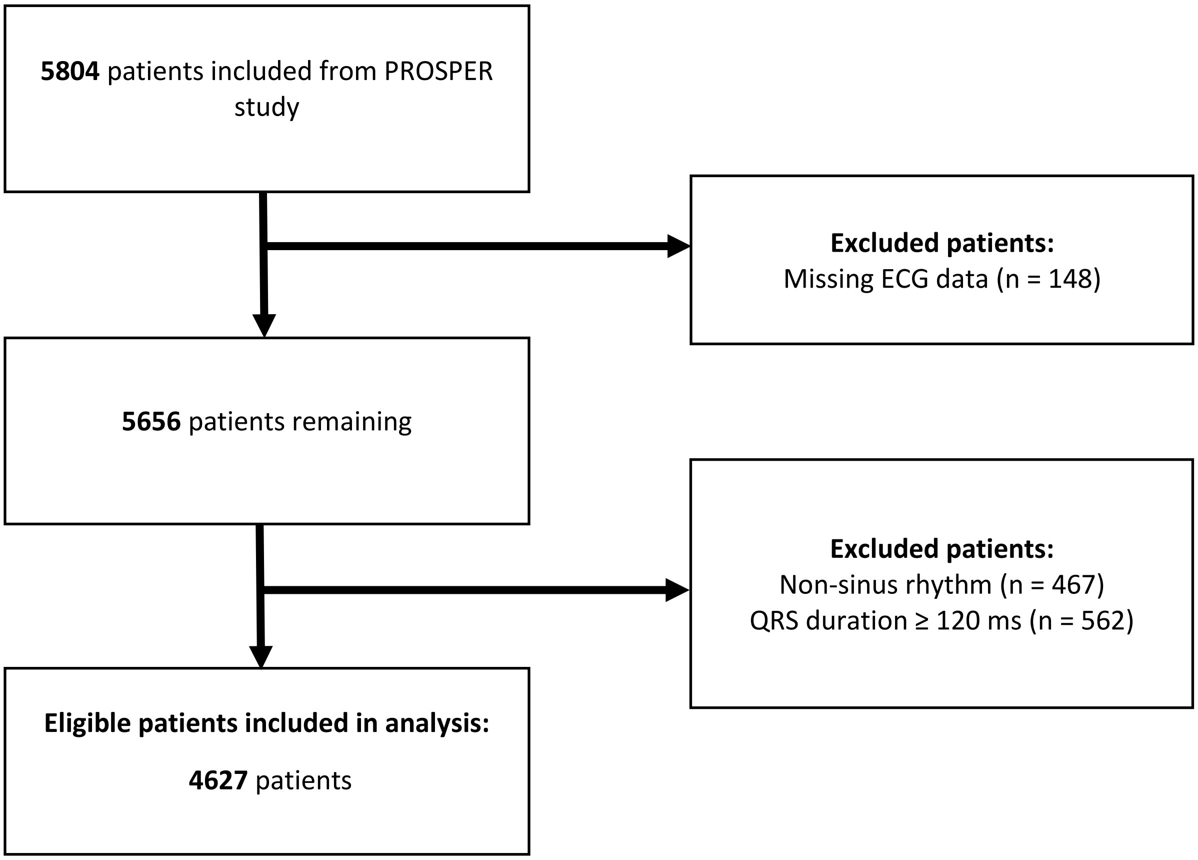

Figure S1. Flow diagram of patient inclusion. Abbreviations: PROSPER, Prospective Study of Pravastatin in the Elderly at Risk; ECG, electrocardiogram.

Table S1. Cross-sectional association at baseline between measures of ventricular de- and repolarization, and brain magnetic resonance imaging (MRI) status parameters (minimally adjusted model).

Brain status	QT (SD) Beta (95% CI)	QTc (SD) Beta (95% CI)	JT (SD) Beta (95% CI)	JTc (SD) Beta (95% CI)	QRS (SD) Beta (95% CI)
**Minimally adjusted**[1]					
White matter hypertensities					
Total lesion volume, ml	–0.74 (–2.18; 0.71)	–0.54 (–1.55; 0.47)	–0.83 (–2.23; 0.57)	–0.61 (–1.62; 0.39)	0.16 (–0.80; 1.12)
Subcortical, mL/y	–0.09 (–0.33; 0.14)	–0.05 (–0.22; 0.11)	–0.13 (–0.35; 0.10)	–0.08 (–0.25; 0.08)	0.05 (–0.11; 0.21)
Periventricular, mL/y	–0.65 (–1.94; 0.64)	–0.49 (–1.39; 0.42)	–0.70 (–1.95; 0.55)	–0.53 (–1.43; 0.36)	0.11 (–0.75; 0.97)
Brain atrophy					
Intracranial volume, ml	15.3 (–1.58; 32.1)	12.6 (0.72; 24.4)	14.8 (–1.55; 31.1)	12.16 (0.40; 23.91)	0.15 (–11.1; 11.4)
Parenchymal volume, ml	8.61 (–4.43; 21.6)	7.48 (–1.68; 16.6)	6.98 (–5.68; 19.6)	6.21 (–2.89; 15.32)	2.09 (–6.61; 10.8)
Of % atrophy (ICV-Par/ICV x100)	0.19 (–0.24; 0.63)	0.13 (–0.17; 0.44)	0.29 (–0.13; 0.71)	0.20 (–0.10; 0.50)	–0.14 (–0.43; 0.15)
Grey matter volume, ml	0.10 (–0.87; 1.06)	0.11 (–0.57; 0.79)	0.02 (–0.93; 0.97)	0.05 (–0.64; 0.73)	0.12 (–0.53; 0.76)
Number of microbleeds	0.36 (0.02; 0.70)	0.18 (–0.06; 0.42)	0.41 (0.08; 0.74)	0.23 (–0.01; 0.47)	–0.08 (–0.31; 0.15)
Number of deep white matter microbleeds	0.04 (–0.05; 0.12)	0.02 (–0.04; 0.08)	0.013 (–0.07; 0.09)	0.01 (–0.05; 0.06)	0.03 (–0.02; 0.08)

[1] = adjusted for age, sex, country, smoking, heart rate (QT, JT, and QRS only). ICV = Intracranial volume, Par = Parenchymal volume

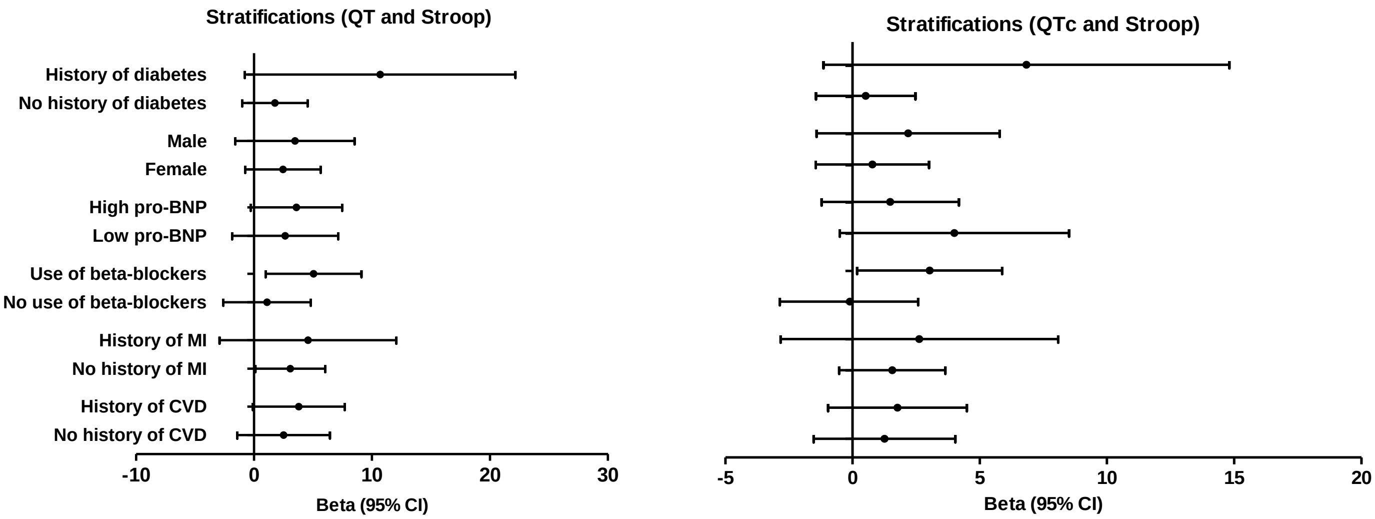

Figure S2. Stratifications for QT and QTc intervals and the Stroop Test (fully adjusted model).

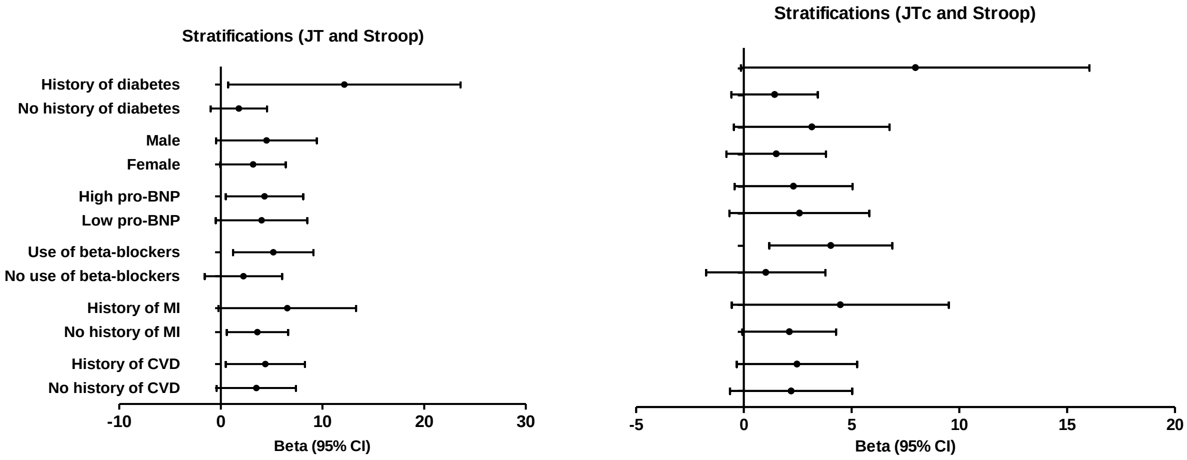

Figure S3. Stratifications for JT and JTc intervals and the Stroop Test (fully adjusted model). BNP=Brain natriuretic peptide. MI=myocardial infarction. CVD=cardiovascular disease.

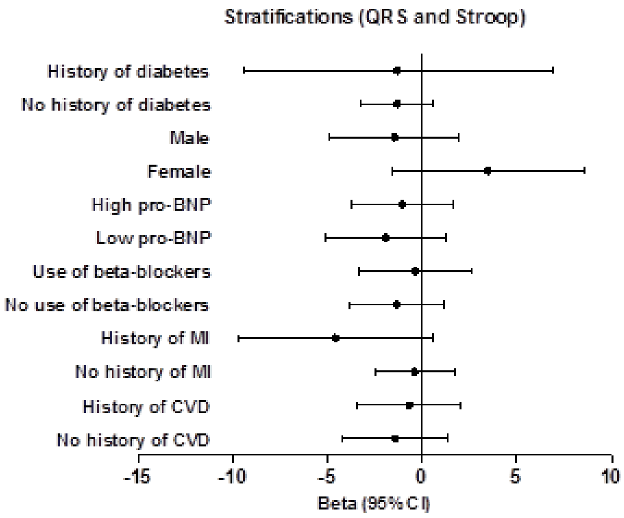

Figure S4. Stratifications for QRS interval and the Stroop Test (fully adjusted model).
